# Automatic Authorship Detection Using Textual Patterns Extracted from Integrated Syntactic Graphs

**DOI:** 10.3390/s16091374

**Published:** 2016-08-29

**Authors:** Helena Gómez-Adorno, Grigori Sidorov, David Pinto, Darnes Vilariño, Alexander Gelbukh

**Affiliations:** 1 Instituto Politécnico Nacional, Centro de Investigación en Computación, Av. Juan de Dios Bátiz S/N, Mexico City 07738, Mexico; sidorov@cic.ipn.mx (G.S.); www.gelbukh.com (A.G.); 2 Benemérita Universidad Autónoma de Puebla, Facultad de Ciencias de la Computación, Av. San Claudio y 14 Sur, Puebla 72570, Mexico, dpinto@cs.buap.mx (D.P.); darnes@cs.buap.mx (D.V.)

**Keywords:** integrated syntactic graphs, textual patterns, authorship attribution, authorship verification, shortest paths walks, syntactic *n*-grams

## Abstract

We apply the integrated syntactic graph feature extraction methodology to the task of automatic authorship detection. This graph-based representation allows integrating different levels of language description into a single structure. We extract textual patterns based on features obtained from shortest path walks over integrated syntactic graphs and apply them to determine the authors of documents. On average, our method outperforms the state of the art approaches and gives consistently high results across different corpora, unlike existing methods. Our results show that our textual patterns are useful for the task of authorship attribution.

## 1. Introduction

A huge amount of information produced on a daily basis is found in various forms of written texts, such as books, journals, technical reports and others. The explosive growth of the number of such documents calls for the development of effective text processing and text mining tools. Better feature extraction techniques for documents is a primary need for many text analysis tasks. Better textual features lead to better performance in tasks, such as document classification, information retrieval, etc.

In traditional approaches, documents are represented with words or *n*-grams using a simple feature vector in the framework of the vector space model (VSM). In this model, the similarity between two documents is estimated using the cosine similarity between the corresponding vectors. This traditional document similarity depends mainly on the similarity of the vocabulary used in the texts. In this way, the semantics and syntax of the documents are ignored when representing the text as a bag of words.

For many problems in natural language processing (NLP), a graph structure is an intuitive, natural and direct way to represent the data. A graph structure is composed of a set of nodes (vertices) and edges (arcs). The edges are pairs of vertices, and depending on the nature of the graphs, these edges can be ordered for directed graphs or unordered for undirected graphs. A graph structure can associate labels or weights to nodes and edges. In computer science, graph structures are often used to model real-world objects. In natural language processing, graphs are used to represent texts during preprocessing steps in tasks, such as textual entailment, word sense disambiguation, etc. [1].

In this paper, we present a generalization of a method for the extraction of text patterns obtained by shortest path traversal over integrated syntactic graphs (ISG) [2]. This method uses a model for representing texts by means of a graph (the ISG) and then extracts features for similarity calculation. The ISG is built using linguistic features of various levels of language description, which provides important information about the texts. We generalize this method with the aim to apply it to various NLP problems.

In particular, we show that patterns discovered with our method can be used in various tasks of the document analysis, such as authorship attribution, authorship verification and question answering, among others. In our previous work, we have evaluated this methodology for question answering (QA) [2]. In this work, we include the results of the experiments carried out for automatic authorship detection (authorship attribution and authorship verification).

Sidorov et al. [3] have shown that syntactic *n*-grams extracted from parsed trees of single sentences are important features for automatic authorship attribution and authorship verification. We show that the same syntactic features, but extracted from integrated sentences (in the ISG) improve the classification of documents in both authorship attribution and authorship verification. A common approach for such problems is to build a classifier using supervised learning. This leads to the need of a large set of documents from each author to be used as training data.

In practice, it is not always possible to obtain a large amount of documents written by the same author. Our methodology, however, is based on a similarity measure calculated with the features extracted from the ISG. It requires as few as one known document per author in order to determine whether an unknown document has been written by the same author.

We show with new experiments that this methodology, which does not require supervised machine learning, obtains good performance for authorship attribution and authorship verification. We also show that our results are promising in comparison with state of the art and baseline supervised methodologies. We analyze the obtained experimental results and discuss the findings.

The paper is organized as follows. Section 2 presents a survey of different text representation schemes proposed in the literature. Section 3 explains in detail our graph-based text representation model. The diverse features included in this representation are discussed and illustrated by examples. Section 4 describes our method for calculating graph similarities based on patterns extracted with shortest path traversal of the graph representation of texts. Section 5 presents the application methodology and the evaluation of the performance of our graph-based similarity for authorship attribution and authorship verification. Finally, Section 6 concludes the paper.

## 2. State of the Art

The vector space model [4] is employed in many text processing problems, such as information retrieval [5], text categorization, authorship attribution, recognizing textual entailment [6,7,8], sentiment analysis [9], etc.

For the construction of a vector space for text representation, one should select the corresponding features and their values. Typical features used for text representation are words, in the bag of words (BoW) model [10], or character *n*-grams [11,12,13]. The values of these features can be Boolean [14], tf-idf (term frequency-inverse document frequency) weights or values based on probabilistic models [15].

These text representations work relatively well for many cases, but since they are based on words that appear at the surface level of texts, they do not reflect the details of meaning implied, or even explicitly expressed, in documents. This sometimes results in failure to detect the similarity of some text passages, which can be due to the variation of words used in the passages. On the other hand, these methods can report non-similar text passages as similar, because of the resemblance of the words used.

An idea to improve the representation of meaning is to represent syntactic relations of words in a text using syntactic *n*-grams [3,16]. Such *n*-grams differ from traditional *n*-grams in that their construction is based on syntactic trees of the sentences. Thus, they consider as neighboring elements to the words or Part-of-Speech (POS) tags that are adjacent in the syntactic tree and not in the linear text sequence. Syntactic *n*-grams can be applied in all NLP problems where traditional *n*-grams are used, say, as features in a vector space model [17,18]. We use a similar idea, to follow the syntactic paths, for the construction of the graph that corresponds to a text. Concept based methods [19,20] have also gained popularity in recent days for text classification.

A graph structure is a natural way to represent texts employed in many research works. A comprehensive study of the use of graph-based algorithms for natural language processing and information retrieval can be found in [1], which describes approaches and algorithmic formulations for: (a) synonymy detection and automatic construction of semantic classes using measures of graph connectivity on graphs built from either raw text or user-contributed resources; (b) calculation of semantic distance in semantic networks, including simple path-length algorithms and more complex random-walk methods; (c) textual entailment judgment using graph-matching algorithms on syntactic or semantic graphs; (d) word sense disambiguation and name disambiguation, including random walk algorithms and semi-supervised methods using label propagation on graphs; and (e) sentiment classification using semi-supervised graph-based learning or prior subjectivity detection with min-cut/max-flow algorithms. Relevant works not mentioned in [1] include the application of graphs in automatic summarization [21], coreference resolution [22], word sense disambiguation [23,24,25], word clustering [26,27] and document clustering [28].

Jin and Srihari [29], proposed a graph-based text representation capable of capturing term order, term frequency, term co-occurrence and term context in documents. This approach tries to discover non-obvious associations between two and more concepts. The authors do not use any linguistic features to construct the graph, in contrast to our approach, which relies on a dependency parser to build the graph representation.

Another graph-based text representation model was proposed by [30] using the word semantic space. The authors also proposed a method of computing text similarity. They did not apply their method to any particular problem of natural language processing; instead, they presented only a theoretical model.

Unfortunately, the majority of the works that use graph-based representations propose ad hoc graph structures that only work with the particular problem with which they deal. In this paper, we propose a general approach that can be used in different NLP problems with minimal changes.

There are few attempts of using deep learning for the authorship attribution task. In [31], the authors propose a framework for learning distributed representations of attributes. The attributes correspond to a wide variety of concepts, such as document indicators (to learn sentence vectors), language indicators (to learn distributed language representations), metadata and additional information about authors (to learn author profile, such as the age, gender and occupation). The framework is evaluated over several tasks: sentiment classification, cross-lingual document classification and blog authorship attribution. For the authorship attribution task, the methodology is evaluated over a corpus of blogs, and the attributes are based only on the author metadata and not on the texts themselves. On the contrary, for our work, we use only the textual information in order to automatically extract a significant representation of documents.

A novel neural network architecture, namely convolutional neural networks (CNN), over word embeddings was presented in [32]. It was evaluated over two datasets, a baseline developed by the author and the dataset of the international workshop on plagiarism detection, author identification and author profiling (PAN) 2012 [33] . For the representation of documents, the author used the set of Google News word vectors trained via the skip-gram method and negative sampling presented in [34,35]. The author used the standard approach for convolutional models (simple concatenation operation) to encode sequences rather than words. The classification is performed via logistic regression and the results show high accuracy over the baseline dataset, but the CNN architecture did not outperform the best method presented at PAN 2012.

There is also related work from the linguistics field. In [36], the authors employed the complex-network approaches for language classification (linguistic typology). They found that complex network parameters can obtain precise language classification, equivalent to contemporary word order typology. In [37], the authors also explore the language classification task. They proposed the substitution of syntactic dependency networks for co-occurrence networks based on parallel text in complex network-based classification. A cluster analysis was performed with appropriate combinations of major parameters of complex networks and correctly-separated Slavic languages from non-Slavic languages, grouping the Slavic languages into their respective sub-branches.

In recent years, complex networks have started to be applied for modeling and analyzing human language [36]. There is already an important amount of research in this area. In [38], the author presents a survey on the complex-network approaches, in three lines of linguistic research. First, the paper explores the characterization of human language as a multi-level system with complex network analysis. Second, the paper presents the application of linguistic networks and their quantitative measures. Third, relationships between the system-level complexity of human language and the microscopic linguistic features are given. The paper concludes with directions for future research on the complex network approach.

Finally, an empirical categorization of Modern Chinese as a multi-level system is presented in [39]. The authors modeled four levels as language sub-systems along the meaning-form dimension based on the same body of corpus data. They performed a topological analysis of the four linguistic networks describing the organizational patterns of the four language sub-systems. The patterns are compared quantitatively, and the interpretation of the results is done from an interdisciplinary perspective.

In previous work [2], we use this representation for question answering, where we try to understand the meaning of a text to be able to answer questions about it. In this work, we propose to extend the ISG representation for a very different problem, automatic authorship detection, where we try to capture only the writing style, and the meaning of the texts should be ignored.

## 3. Representation of Texts Using Integrated Syntactic Graphs

The ISG textual representation model was proposed by [2] with the aim of integrating into a single data structure multiple linguistic levels of natural language description of a given document. This model is able to capture most of the features available in the document, from the morphological to the semantic and discursive levels. By including lexical, syntactic, morphological and semantic relations in the representation, the model is capable of integrating in the ISG various text units, such as words, phrases, clauses, sentences, etc.

In this paper, we consider also the lexical level, in addition to the traditional levels of analysis, like morphological, syntactic and semantic levels. We made this decision for convenience when we are treating the words without the context. Note that in linguistics, there are several levels of language analysis, like phonology, morphology, syntax, semantics, pragmatics and discourse. They represent approximations for language analysis, and there are no strict boundaries between these levels; they are more like the points of view of a researcher for the purposes of analysis; say, semantics (meaning) is also present in morphology or syntax; or say, there is also morphonology, which is somewhere between phonology and morphology.

A detailed description of this representation model can be found in [2]. However, we summarize here some of its characteristics for the sake of completeness. The ISG is built of the following language description levels:

- **Lexical level**: ISG is able to represent lexical items, such as words, without reference to the sentence in which they occur.
- **Morphological level**: At the morphological level, ISG deals with the identification, analysis and description of the structure of the given language’s morphemes, besides other linguistic units, such as roots, stems, affixes and parts-of-speech (POS) tags.
- **Syntactic level**: At the syntactic level, ISG stores rules and principles that govern the sentence structure. Though there exists different syntactic formalisms, [2] suggests to use dependency formalism. This formalism is also used by other authors for extracting syntactic *n*-grams [3,16].
- **Semantic level**: At the semantic level, ISG introduces the meaning of a sentence or a text into the graph. There exists a large body of literature on linguistic semantics. We are particularly interested in paradigmatic semantic relations. A number of paradigmatic semantic relations were identified in different disciplines, such as linguistics, logic and cognitive psychology [40]. The most widely-used paradigmatic semantic relations are antonymy, synonymy, class inclusion, part-whole and case [41].

Note that we included POS tags in the morphological category, because labels usually codify grammatical categories, such as number and gender. For example, the EAGLES (Expert Advisory Group on Language Engineering Standards) POS tag sets (https://talp-upc.gitbooks.io/freeling-user-manual/content/tagsets.html) “consist of variable-length labels where each character corresponds to a morphological feature”. Additionally, Pierre M. Nugues, in his book on natural language processing [42] in Chapter 5 entitled “Words, Parts of Speech, and Morphology” states that “we can divide the lexicon into POS, that is, classes whose words share common grammatical properties”. It is logical that POS are properties of words; they express grammar classes, like noun, verb, etc., and grammatical categories, like tense, time, number, etc. They do not express relations of a word with other words, which is the principal focus of syntax.

### Construction of the Integrated Syntactic Graph

Construction of the ISG involves analyzing all sentences of the text. We apply the Stanford dependency parser [43] in order to obtain the parse trees of the sentences. The nodes of all trees with the same label, such as the nodes corresponding to different occurrences of the same word type (lemma and POS tags) are then identified, so that by each word type, there is only one node in the ISG. Since the syntactic tree of each sentence contains the root node and all root nodes are collapsed into one node of the ISG, the resulting graph is connected. In our implementation, we construct the ISG in an incremental way. After analyzing each sentence, we add to the ISG the corresponding new nodes or new arcs between nodes if they did not exist in it before.

**Figure 1:**
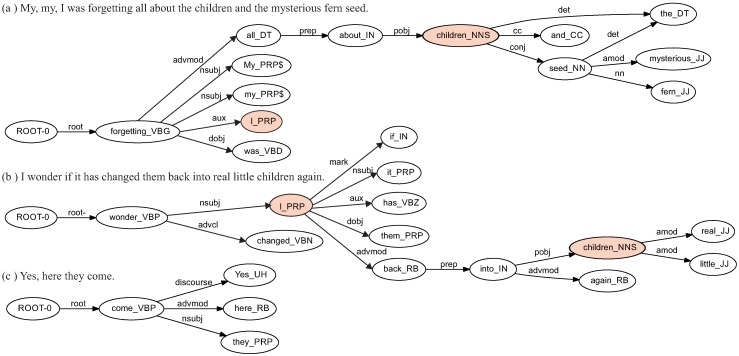
Dependency trees of three sentences of the target text using word_POS combination for the nodes and dependency labels for the edges.

**Figure 2:**
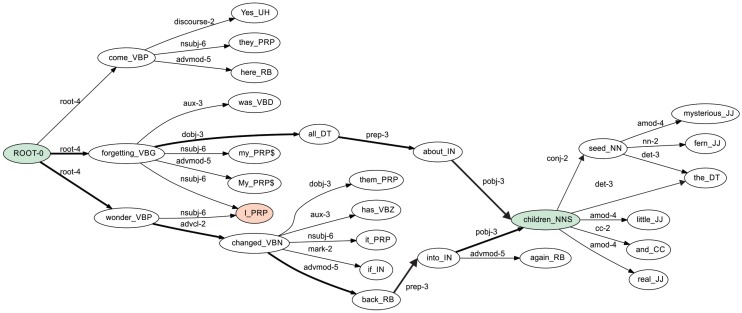
The integrated syntactic graph for the three sentences shown in Figure 1.

In Figure 1, the dependency trees of three sentences are shown; each node of the tree is augmented with other information, such as the combination of lemma (or word form) and the POS tag. The collapsed graph of the three sentences is shown in Figure 2. Each edge of this graph contains the dependency tag together with a number that indicates the frequency of that dependency tag plus the frequency of the pair of nodes, both calculated by using the occurrences of the dependency trees associated to each sentence.

In our experiments, words in the ISG differ only by their POS tag, i.e., if the word is used as a noun, a verb, an adjective, etc. Words with the same POS tags, but different meanings are collapsed into one node in the graph. However, the ISG formalism allows for separating words by their senses. For this, at the preprocessing stage, a word sense disambiguation step is to be added before collapsing of the nodes. For question answering, we previously conducted anaphora resolution on the texts. However, in the case of author attribution, we believe that there is no need to introduce this preprocessing, since syntactic features should be more important for this problem than lexical and semantic ones [3,44].

**Figure 3:**
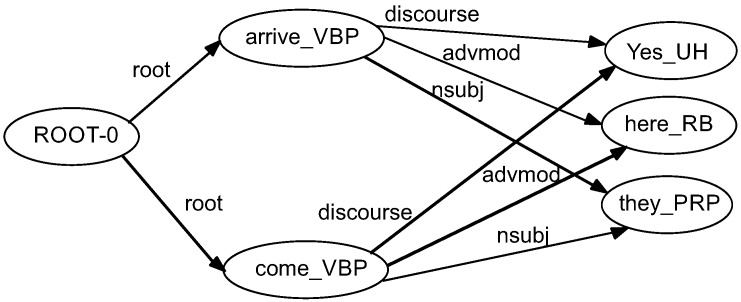
Syntactic tree with the synonym expansion of a sentence: “Yes, here they come”.

In order to extract semantic relations between words in a text, we used the WordNet taxonomy [45]. Figure 3 illustrates the manner in which we integrated synonyms into the graph-based representation. Other semantic relations also can be included in the graph. For instance, the node *come_VBP* is expanded with a synonym, *arrive_VBP*. The new added node *arrive_VBP* inherits the relations of the original nodes, with the same direction and labels on the arcs.

The inclusion of the semantic expansion process is optional, and it can be helpful for some tasks. For this paper, we did not perform semantic expansion, but it is part of our future work. Below, we describe how the textual patterns are extracted to be used in language understanding problems. In this paper, we show this on the example of automatic authorship attribution and verification.

## 4. Extraction of Textual Patterns from ISG

In this section, we present a textual feature extraction technique based on the graph representation of a given text. Recall that the graph can represent a sentence, a paragraph, a document or even a collection of documents. We assume that the graph uses the representations discussed in the previous section. Our technique allows finding patterns in the graph by counting text elements (lemmas, POS tags, dependency tags), while shortest paths are traversed in the graph.

Consider the ISG shown in Figure 2. There are two paths connecting the node *ROOT-0* with the node *children_NNS*. We take the shortest path that has the following features at different language description levels:

- **Lexical level**: *ROOT-0*, *forgetting*, *all*, *about*, *children*;
- **Morphological level**: *VBG*, *DT*, *IN*, *NNS*;
- **Syntactic level**: *root*, *dobj*, *prep*, *pobj*.

These textual patterns can further be used as features in a particular NLP problems. Thus, a text represented by a graph provides a set of features for each of the shortest paths found in this graph, which can later be used, for example, for constructing a vector space model representation of the document. Actually, given two or more graph-based representations of texts, we can compare these graphs, and consequently the documents, using the textual patterns extracted with our technique.

In Table 1, we present an example of the features extracted using the shortest paths in the graph. Each row represents one path. The labels *VBG*, *DT* and *PRP* correspond to POS tags identifying verbs, determinants and pronouns respectively. The labels *dobj*, *nsubj* and *pobj* correspond to dependency tags identifying relationships between pair of words, specifically, the relationships are: direct object, nominal subject and object of a preposition, respectively. The paths are extracted from the ISG shown in Figure 2, considering the pairs: (*ROOT-0*, *children_NNS*), (*ROOT-0*, *I_PRP*) and (*ROOT-0*, *them_PRP*). For example, Row 2 indicates the path to reach the node *I_PRP* from *ROOT-0*. In Column 2 of Row 2, there is a number 1 indicating the presence of the word *forgetting* in the path. Row 3 also indicates the path to reach the node *I_PRP* from *ROOT-0*, but this is another path. In Column 2 of Row 3, there is a number 0 indicating the absence of the word *forgetting*. Please refer to Figure 2 to observe these two paths. In general, the number of pairs considered as the initial and final node can vary. For instance, considering all combinations for the *n* nodes in the graph, the time complexity is $O(n^{2})$. If we fix the initial or the final node, the time complexity is O(n). Different strategies can be applied depending on the particular text processing problem. In our case, we fix the initial node of the ISG, i.e., the *ROOT* node.

The procedure starts by selecting the root node of the graph as the initial node for the path traversal, whereas the final nodes correspond to the remaining nodes of the graph reachable from the initial node. When comparing two graphs with this technique, the final nodes represent the words from the smallest graph. We used the Dijkstra’s algorithm [46] for finding the shortest path between the initial and the final nodes. While traversing the paths, we count the occurrences of all multi-level linguistic features considered in the text representation.

For example, given the pair (*ROOT-0*, *children_NNS*), there are two ways to reach the node *children_NNS* from *ROOT-0*, but the shortest one is *ROOT-0*, *forgetting_VBG*, *all_DT*, *about_IN*, *children_NNS*. We gather the linguistic information from that path, storing it in a feature-value structure. The obtained textual features are the complete linguistic information available in the graph, i.e., words (or lemmas), POS tags, dependency tags, etc.

The decision of using only shortest paths for the graph traversal is based on our previous work [47], where we observed that the use of the shortest path for traversing the graph not only results in a very fast approach for comparing features in a graph representation, but also yields good results. In [47], we considered the possibility of the existence of more than one shortest path between two nodes. Therefore, experiments were conducted using all shortest paths instead of only one. When we use all shortest paths for question answering, our method answers 42% of the questions correctly for one test dataset, the QA4MRE (Question Answering for Machine Reading Evaluation) 2011 data set and, 27% for the other test dataset, the QA4MRE 2012. The same result is obtained for the QA4MRE 2011 data set when using only one path, obtained with the Dijkstra’s shortest path algorithm, and a slightly lower result of 24% for the QA4MRE 2012 dataset.

We observed that one shortest path was enough for identifying the important features and was less time consuming. Therefore, instead of considering all of the shortest paths between two given nodes in this work, we decided to employ only one short path, namely Dijkstra’s algorithm.

The textual patterns extracted from ISGs can be used for different applications. One of the most common uses is to calculate the similarity between two different texts; this will be discussed in the next section. Another use for these features is to construct syntactic *n*-grams [48] while traversing the paths and to use them as features in a VSM for a classification algorithm. When we traverse the different paths in the graph, we transform each path into a syntactic *n*-gram [48] that represents the path. Each syntactic *n*-gram is treated as a single feature in the VSM, and it can be represented with different weighting schemes (frequency, Boolean, etc.)

### 4.1. Use of ISG Textual Patterns for Calculating Text Similarity

In order to compute text similarity, we first build the ISG for two compared documents and then obtain the textual patterns for each document, which gives a set of *m* feature vectors $\vec{f_{t,i}}$ for each text *t*.

The idea is to search for occurrences of the features of a test document (i.e., a document of unknown authorship) in a much larger graph (a graph of documents of known authorship). In a graph corresponding to one author, we collapse all documents written by the author, and therefore, it contains all of the characteristics of this specific author.

Thus, the unknown author’s graph $D_{1}$ is represented by *m* feature vectors $D_{1}=\left\{\vec{f_{D1,1}},\vec{f_{D1,2}},\cdots ,\vec{f_{D1,m}}\right\}$ and the known author’s graph $D_{2}$ by feature vectors $D_{2}=\left\{\vec{f_{D2,1}},\vec{f_{D2,2}},\cdots ,\vec{f_{D2,m}}\right\}$. Here, *m* is the number of different paths that can be traversed in both graphs, using the *ROOT-0* node as the initial node, and each word appearing in the unknown author’s graph as the final node.

Once we obtain the vector representation of each path in a pair of graphs, we adapt the cosine measure for determining the similarity between the unknown document $D_{1}$ and the known document $D_{2}$, using cosine similarities between paths:

$$\begin{array}{cc} Similarity(D_{1},D_{2}) & =\sum_{i=1}^{m}Cosine(\vec{f_{D_{1},i}},\vec{f_{D_{2},i}})\\ & =\sum_{i=1}^{m}\frac{\vec{f_{D_{1},i}}\;\;\;\cdot \;\;\;\vec{f_{D_{2},i}}}{\left|\right|\vec{f_{D_{1},i}}||\;\;\;\cdot \;\;\;||\vec{f_{D_{2},i}}\left|\right|}\\ & =\sum_{i=1}^{m}\frac{\sum_{j=1}^{\left|V\right|}\left(f_{(D_{1},i),j}\times f_{(D_{2},i),j}\right)}{\sqrt{\sum_{j=1}^{\left|V\right|}{\left(f_{(D_{1},i),j}\right)}^{2}}\times \sqrt{\sum_{j=1}^{\left|V\right|}{\left(f_{(D_{2},i),j}\right)}^{2}}}, \end{array}$$

where *V* is the total number of linguistic features.

## 5. ISG Textual Patterns for Automatic Authorship Detection

In this section, we present the application of the textual patterns extraction based on ISG for two problems associated with automatic authorship detection: authorship verification and authorship attribution.

We evaluated two approaches for solving both problems using our methodology. Both approaches are explained below:

- **Profile-based approach:** Construct one ISG that includes all training documents for each author in the training set. In this case, each graph represents one author. All of the linguistic textual patterns proposed in the previous section are generated as described above. As a result of this process, each author is represented by an ISG. The test documents are represented with the ISG, as well, but each document has a corresponding graph.
- **Instance-based approach:** Construct one ISG for each document in the training set for all authors. In this case, each graph represents one document. All of the linguistic textual patterns proposed in the previous section are generated as described above. As a result of this process, each document of all authors is represented by an ISG. In the same way, each test document is represented with a corresponding graph.

### 5.1. Authorship Verification

Authorship verification is a problem related to automatic authorship detection and can be described as follows. Given a set of documents written by a single author and a document in question, the goal is to determine if this document were written by this particular author or not [49]. It is a variant of the general authorship attribution problem with the binary classification of the authors: yes or no.

For authorship verification, we performed the evaluation over the corpus presented in the international workshop on plagiarism detection, author identification and author profiling (PAN’13 and PAN’14) [50,51]. The evaluation corpus for English language in PAN’13 comprises 30 problems, and the English corpus in PAN’14 is divided in essays and novels with 200 problems each. Each problem contains a set of documents of known authorship (the same author) and one document of questioned authorship. The task consists of determining, for each problem, whether or not the test document is written by the given author.

This task is more complex than authorship attribution, because the training set is small in the evaluation corpus (PAN’13), and it can be composed of only one document. Therefore, it is difficult to solve it as a supervised classification problem where usually a larger training set is needed. Authorship verification methods can be divided in two categories, intrinsic or extrinsic. Intrinsic methods only use the known texts and the unknown text of each problem to decide whether they are written by the same author or not. Intrinsic methods do not need any other texts by other authors. On the other side, extrinsic methods need additional documents by other authors found in external resources and use them as negative samples for each problem. The majority of the methods presented in PAN’14 falls into the intrinsic category. However, the winner system of PAN’13 belongs to the extrinsic category [50].

We propose two methods for the authorship verification problem, one for each of the above categories, knowing that each problem has 10 documents or less (possibly as few as one document) by one “known-author” and a “questioned” document (also named “unknown-author” document). Both methods are explained below:

- **Extrinsic approach**: For each problem, we concatenate the “known-author” documents and represent them with an ISG as described in the previous section. After this, the “unknown-author” document of each problem is individually represented with an ISG using the same features. In this way, we obtained one ISG for each “unknown-author” document. In order to identify if an “unknown-author” document corresponds to the author of the problem in question, we calculate the similarity of that “unknown-author” document (graph) with each “known-author” document (graph) of all problems. If the “questioned” graph is more similar to the graph of the problem to which it belongs, then the answer is “yes”, i.e., it belongs to this author. However, if the “unknown-author” document is more similar to a document of another problem, then the answer is “no” (it does not belong to this author).
- **Intrinsic approach**: For each problem, we concatenate the “known-author” documents and represent them with an ISG as described in the previous section. After this, the “unknown-author” document of each problem is individually represented with an ISG using the same features. In this way, we obtained one ISG for each “unknown-author” document. In order to identify if an “unknown-author” document corresponds to the author of the problem in question, we calculate the similarity of that “unknown-author” document (graph) with the “known-author” document (graph) of that problem. If the similarity is greater than a predefined threshold, then the answer is “yes”, i.e., it belongs to this author. However, if the similarity is lower than the predefined threshold, then the answer is “no” (it does not belong to this author). The threshold is currently obtained from the training set by averaging the similarities scores of all problems. The threshold is fixed for the complete test corpus.

It is important to remember that this methodology does not use supervised machine learning, and it does not need to use the training corpus. Thus, we use only the documents of known and unknown authorship of the test set for the evaluation. We only use the training set for tuning the threshold of the intrinsic approach. The evaluation results are given in Section 5.5.

### 5.2. Authorship Attribution

Authorship attribution aims at identifying the author of a given text. It is a classification problem: given a set of documents for various authors and a set of documents with unknown authors, it is necessary to detect their authors, i.e., to choose the class that corresponds to the authors of each unknown document. To accomplish this task, it is necessary to identify characteristics (features) or profiles that correspond to the target authors. This is a difficult task because writing styles between authors are often similar. Various techniques were developed for solving this problem. Stamatatos presents an interesting survey about this topic in [44]. In [52], the authors present a reproducibility study on author identification research. They selected fifteen of the most influential papers for author identification and reimplemented them. They evaluated each methodology in three corpora and showed that only four out of fifteen (4/15) approaches are stable across corpora. Moreover, the approaches that employ character features seem to be the most effective ones for this problem.

For the authorship attribution problem, we used the corpus C10 presented in [53,54]. This corpus is a subset of the Reuters Corpus Volume 1 (RCV1) [55]. It has documents written by 10 authors, and there are 50 documents for each author for training and testing.

Our method is based on the idea of representing both the training and the test sets with the ISG and, then extracting the textual features directly from the graph representation. For this task, we only follow the profile-based approach. In order to identify the author of a given test document, we calculated the similarity between the ISG that represents the test document and each one of the ISGs representing the ten authors. Recall that in the profile-based approach, we build one graph per author.

Finally, the training graph that obtains the higher degree of similarity with the test document corresponds to the author of the test document. The evaluation results are given in Section 5.4.

### 5.3. Experimental Setup for Authorship Attribution and Verification

We executed experiments with different feature sets. Table 2 shows the features extracted from the ISG and remarks about those features that were used for each configuration. The same feature sets were used over three evaluation corpora: C10 (for authorship attribution), PAN’13 and PAN’14 (for authorship verification).

There are two concepts to be considered in Table 2, the features extracted from the ISG and the features included in the ISG representation. Table 2 shows the different set of features that we extracted from the ISG for these experiments. In the following paragraph, the structure of the graph is explained. There are differences when we include the words in the nodes and when we use lemmas instead of words. When using lemmas, the graph is smaller than when using the word form as appearing in the texts.

The *MorSyn* feature set includes the extraction of POS tags of the nodes of the graph, as well as dependency tags, which represent the relations between each pair of nodes (vertices). Note that in this feature set, we do not use lemmas in graph nodes and do not consider frequencies for the vertices. The *LemFreq* feature set also includes the extraction of POS tags of the nodes of the graph, as well as dependency tags. We replace the words by their lemmas and add the frequency count of the vertices (initial node to final node + dependency tags) in the same way as is explained in Section 3.1. The rest of the feature sets are named in a similar way according to the features they extract.

Following previous research [56], we also added counts of vowels (Voc) and counts of word suffixes (Suf) for the *LemFreqLexMorVocSuf* feature set. While traversing the path, we verify in every node the vowels and suffixes of the word or lemma represented in this node and use their counts as additional features. For example, in a given path containing the following words, introduction, relationship and facility, the vowels in these words are *iouio*, *eaioi* and *aiiy*, whereas the suffixes are *-tion*, *-ship* and *-ity*.

### 5.4. Results for Authorship Attribution

**Figure 4:**
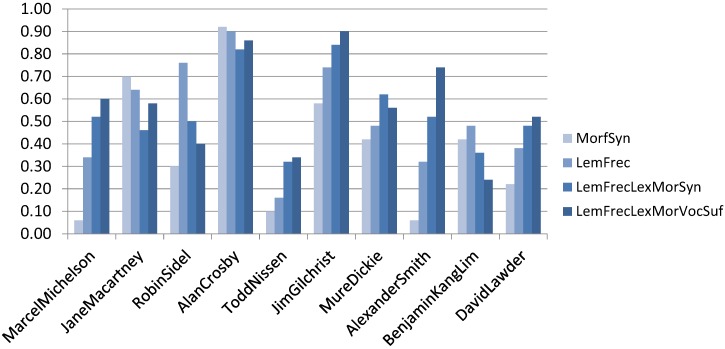
Accuracy for each of the ten authors.

For the evaluation procedure, we mainly used the accuracy measure. However, we also report precision, recall and F1-score in Table 3. Figure 4 depicts the results obtained for each one of the ten authors of the C10 Corpus using the different feature sets.

As one can see, there are authors that were benefited by the use of additional components in the ISG, for example lemmatization and POS tags. However, there are also authors who did not. We consider that this behavior is due to how specific or how consistent the writing style of some authors is. This assumption will be verified in future work. Some authors have a writing style that is much easier to be identified and modeled, for example, Alan Crosby. In these cases, the inclusion of new characteristics did not contribute to better attribution.

In Table 3, we present the summary of the results obtained using each feature set in the C10 corpora using the profile-based approach. Table 4 presents the results of the best methods reported in the literature for this corpus [53,57]. The *LemFreqLexMorVocSuf* feature set obtained the best performance for our methodology, achieving an accuracy of 68.0%.

The results obtained by [53,57] for the C10 corpus are better than ours, but we must take into account that they use a supervised approach and we do not use supervised machine learning. In [53], the authors use an SVM algorithm to perform the classification using the 2,500 most common three-grams used across the training documents and report an accuracy of 80.8%. In [57], the authors report an accuracy of 78.2% and 75.0% using the BOW representation scheme calculated over words and character *n*-grams, respectively, with the SVM algorithm to perform the classification process. In the same work, Escalante and Solorio outperform the best reported result for this dataset, achieving an accuracy of 86.4% with the local histograms of character *n*-grams representation. However, in the reimplementation work of [52], the best approach achieved only 76.6% accuracy on the C10 corpus. As it can be seen, our results are lower than the state of the art for this corpus. However, it must be taken into consideration the fact that our methodology does not use any classification algorithm, and it only relies on a similarity measure. We consider that our approach achieves good results (not the best), and it is easy to apply in practice, given that it does not need a large set of labeled data.

Even if other authors have obtained very good performances in authorship attribution (using the C10 collection) by employing simple models, such as most frequent character *n*-grams or histograms of character *n*-grams, our model is still interesting, because it accepts other types of features different from the sequence of characters for representing the texts. We consider that in some problems, for example authorship attribution, a sequence of character *n*-grams is a simpler and effective way of representing the target text for discovering the writing style of an author. However, this representation requires a machine learning algorithm, therefore, a large set of labeled instances is needed.

### 5.5. Results for Authorship Verification

In Table 5, we present the summary of the results obtained using each feature set in PAN’13 English corpus using the profile and instance-based approaches. The *LemFreqLexMorVocSuf* feature set using the profile-based approach obtained the best performance. The results obtained by the instance-based approach are lower than the profile-based; therefore, we believe that when we concatenate all of the documents for an author, the features are potentiated. When the documents are separated into individual graphs, important information is lost.

In the PAN’13 competition, 18 teams submitted their software for author verification [50]. The random baseline system proposed by the task organizers achieved an accuracy of 50.0%. The best ranked system developed by Seidman [58] achieved an accuracy of 80.0% for the English dataset; they use the impostor’s method [59] for comparing the similarity between the target document (unknown document) with respect to both a known document and a set of external (impostor) documents, randomly selecting a number of features from a feature pool. This process is repeated a parameterizable number of times. If they find that the unknown document is more similar to the impostors than the known document, then it does not belong to the author of the given problem. The result obtained by [58] was outperformed by our methodology, which obtains an accuracy of 83.3% over the same set.

We computed a statistical significance test to measure the difference of performance between our methodology and the best performing systems of PAN’13 and PAN’14. The organizers of PAN provided us the output of the participating systems. We used the approximate randomization testing [60] implemented by Vincent Van Asch (http://www.clips.uantwerpen.be/scripts/art). We did a pairwise comparison of the accuracies of our results against the best results of PAN for the corresponding datasets. The system is considered to be significantly different with *p* < 0.05. The results of the significance test against Seidman’s work [58] gave p=1.0, which indicates that there is no difference between the performance of the systems.

In Table 6, we present the summary of the results obtained using each feature set in the PAN’14 English corpus following an intrinsic approach. The *LemFreqLexMorVocSuf* feature set using the profile-based approach obtained the best performance; for this corpus, we did not evaluate the instance-based approach. In the PAN’14 competition, 13 teams submitted their software for author verification [51]. The baseline system proposed by the task organizers achieved a score of 53.0% for English essays and 44.5% for English novels. The best ranked system for English essays achieved a score of 71.0% [61] and for English novels a score of 71.5% [62]. In Table 6 .

The methodology presented in [62] obtained 71.5% precision for the English novel corpus. It was the only system that outperformed our methodology, which obtained a precision of 66.5% over the same set. For the English essays, our methodology obtained a score of 60.0%, which is a low result, but it overcame the baseline score in PAN’14 for this corpus. From these results, we can conclude that, for the author verification task, our methodology works better when the input texts are longer. In this case, the English novel corpus had longer texts than the English essays.

We took the best configurations (*LemFreq* for English novels and *LemFreqLexMor* for English essays) in order to evaluate if the difference of performance between both of them is statistically significant from the best systems in PAN’14. The results of the significance test against Frèry’s work [61] for the essays dataset gave p=0.1, which indicates that there is no difference between the performance of the systems. The results of the significance test against Modaresi’s work [62] for the novels dataset gave p=0.3, which also indicates that there is no difference between the performance of the systems. In both cases, our model obtained lower results than the best systems of PAN’14, but the significance test indicates that these differences are not significant.

From the results presented in this section, we can draw several conclusions. First, our methodology works better with a profile-based approach rather than an instance-based approach for corpora with large documents (e.g., English novels). This assumption is obtained from the PAN’14 experiments, where we obtained the best results when concatenating all of the documents of the same author in only one graph that contains all known features of such an author. Second, our methodology works better with a corpus with large documents (in terms of accuracy), although this is much more time consuming. For PAN’14, we performed a “filtered” extrinsic approach, due not only to the extension of the document, but the quantity of the problems. Both English essays and novel corpora were composed of 200 problems. This means that if we wanted to compare each of the “unknown problems” against the graphs of the “known authors”, there would will be 40,000 comparisons. This issue was solved by randomly selecting a sample of 20 authors for the comparison. For the English novel corpus of PAN’14, we did not perform the instance-based approach, because there is only one known document per problem in the entire corpus. Therefore, for the English novel corpus, the instance-based approach is equivalent to the profile-based approach.

## 6. Conclusions

In this paper, we extended the textual pattern extraction process over ISGs and their use for automatic authorship detection, namely authorship attribution and authorship verification. We defined a manner of representing lexical, morphological, syntactic and semantic relations as textual features of a document during its conversion into a single graph of the entire document (the ISG). The graph-based representation can contain words, lemmas, POS tags, syntactic tags and paradigmatic semantic relations, such as synonymy, hypernymy or antonymy. The capability of integrating multiple levels of natural language description in a single structure makes it a richer and more expressive representation than others reported in the literature.

We obtained textual patterns by traversing shortest paths in the ISG, determining the aforementioned linguistic features. Further, we applied the cosine measure, calculated over shortest paths using these textual patterns for a pair of graphs, for determining the similarity between every pair of documents analyzed in the experimental results.

Although we could consider other kinds of paths, i.e., different than the shortest ones, we already evaluated their use in previous experiments for question answering [47]. For that reason, we conclude that the shortest paths contain the most representative contextual information for the words represented in the initial and final nodes.

The feature vectors extracted from the ISG can be used in several ways, for instance, for building rich syntactic *n*-grams, by introducing them into machine learning methods, or as representative vectors of a document collection. In this particular research work, we employed them for graph similarity calculation.

In order to determine the performance of the proposed textual patterns, we conducted experiments for automatic authorship attribution and authorship verification. We showed that these patterns, used as features, allow finding the correct author of a document (authorship attribution) with a precision of 68.0% for the C10 corpus, which is higher than a cosine baseline approach. We also performed experiments for the PAN’13 corpus (authorship verification), obtaining higher precision (83.3%) than the best method reported in the competition working notes (80.0%) [50], for the English language. From the results obtained with the PAN’14 corpus, we can observe that they are comparable with the state of the art. Although our results only outperform other approaches in some cases, our methodology is consistent across different corpora. In [52], it was shown that only four out of 15 methodologies for authorship attribution were stable across corpora; the methodologies that achieve the best performance in one corpus failed on the others.

From the obtained results, we can draw several conclusions and answers for some research questions about ISGs. First, we observed that for automatic authorship detection, the best results are achieved while more features are added to the graph. In all of our experiments, we started with a basic graph composed of only POS tags and dependency tag features. Thereafter, we included the frequency count, and it improved the results because the shortest path algorithm gives preference to the lowest frequency paths, i.e., it prefers the edges with lowest weight. For this reason, we believe that the shortest paths represent better the writing style of an author.

Semantic features are proven to be helpful when they are used in combination with other kinds of features, such as syntactic and morphological ones [44]. Therefore, for future work, we are planning to evaluate the semantic relationships expansion in the ISG for the automatic authorship detection.

In general, the textual patterns obtained from the ISGs can be applied in different problems of NLP, as was shown in this paper. An important advantage of our methodology is that it does not need a large training set in order to build a model. Finally, to employ this approach to various NLP problems, it is important to: (1) evaluate if the task can be solved with a similarity-based approach and if it is necessary to reformulate the task, as we did with the authorship attribution task; (2) analyze which kinds of features are traditionally more effective in that task in order to add this features to the ISG; and (3) identify which document will become the query document (in our case, the unknown-author documents) and which one the base document (in our case, the known-author documents).

As future work, we will evaluate the usefulness of the textual patterns in other NLP problems, such as textual entailment, textual semantic similarity, automatic classification of tweets, etc. We will also evaluate different configurations of parsing tools for the construction of the graph-based representation of the texts. Furthermore, we will propose a manner of integrating other types of text tagging (including named entity recognition, polarity detection, among others) into the ISG. Finally, we consider it important to evaluate our methodology for automatic authorship detection in other corpora, such as C50 [53], Raghavan et al. [63] and PAN’15 [64].

## Figures and Tables

**Table 1:** Representation of a text using the feature extraction technique.

Initial Node to Final Node	Lexical Features				Morphological Features				Syntactic Features			
	*forgetting*	*all*	⋯	*I*	*VBG*	*DT*	⋯	*PRP*	*dobj*	*nsubj*	⋯	*pobj*
*ROOT-0* **to** *children_NNS*	1	1	⋯	0	1	1	⋯	0	1	0	⋯	1
*ROOT-0* **to** *I_PRP*	1	0	⋯	1	1	0	⋯	1	0	1	⋯	0
*ROOT-0* **to** *I_PRP*	0	0	⋯	1	0	0	⋯	1	0	1	⋯	0
⋮			⋮				⋮				⋮	
*ROOT-0* **to** *them_PRP*	0	0	⋯	0	0	0	⋯	1	1	0	⋯	0

**Table 2:** Setup used in the experiments.

	Features						
**Name of Feature set**	**Words (count)**	**POS tags (count)**	**Dependency tags (count)**	**Combination of Vowels (count)**	**Permutation of Vowels (count)**	**Suffixes (count)**	**Synonym Expansion**
*MorSyn*		✓	✓				
*LemFreq*		✓	✓				
*LemFreqLexMorSyn*	✓	✓	✓				
*LemFreqLexMorVocSuf*	✓	✓	✓	✓	✓	✓	

**Table 3:** Results for authorship attribution using the C10 corpus. The best performing feature set is in bold.

Feature Set	Accuracy	Precision	Recall	F1-score
*MorSyn*	43.0	63.0	36.4	46.1
*LemFreq*	55.0	65.2	51.2	57.4
*LemFreqLexMor*	67.0	71.2	66.8	68.9
*LemFreqLexMorVocSuf*	**68.0**	72.1	68.0	70.0
*CosineBaseline*	65.0	65.2	65.6	65.4

**Table 4:** Results for authorship attribution with supervised-based methods, using the C10 corpus. The best performing supervised approach is in bold.

Supervised Methods	Accuracy
BOW (words)	78.2
BOW (characters)	75.0
Char trigrams	80.8
LH Char 3-grams [57]	**86.4**

**Table 5:** Results for authorship verification using the extrinsic and intrinsic approaches for the PAN’13 English corpus. The best performing feature sets are in bold.

Feature Set	Instance Based	Profile Based	
	Extrinsic	Extrinsic	Intrinsic
*LemFreq*	53.3	73.3	76.6
*LemFreqLexMor*	66.6	**83.3**	76.6
*LemFreqLexMorVocSuf*	66.6	**83.3**	76.6
Random Baseline	−	−	50.0
Best PAN’13 System	80.0	−	−

**Table 6:** Results for authorship verification using the instance-based and profile-based approaches with extrinsic and intrinsic methods for the PAN’14 English corpus. The best performing approach for each corpus is in bold, while our best results are underlined.

	Profile Based			
	**English Essays**		**English Novels**	
	**Intrinsic**	**Extrinsic**	**Intrinsic**	**Extrinsic**
*LemFreq*	48.8	51.5	66.5	60.0
*LemFreqLexMor*	48.5	52.0	66.5	61.0
*LemFreqLexMorVocSuf*	48.0	49.5	62.5	54.87
	**Instance Based**			
*LemFreq*	52.7	57.0	−	−
*LemFreqLexMor*	52.2	60.0	−	−
*LemFreqLexMorVocSuf*	51.2	53.0	−	−
Baseline System	53.0		44.5	
1st. PAN’14 System	**71.0**		**71.5**	
2nd. PAN’14 System	65.5		65.0	
